# The Effect of Artificial Ageing on the Changes in Selected Properties of Organic Coated Sheets

**DOI:** 10.3390/ma17163891

**Published:** 2024-08-06

**Authors:** Lydia Sobotova, Janette Brezinova, Miroslav Badida, Miroslava Badidova, Barbara Ciecinska, Alica Maslejova

**Affiliations:** 1 Department of Business Management and Environmental Engineering, Faculty of Mechanical Engineering, Technical University of Kosice, 040 01 Kosice, Slovakia; miroslav.badida@tuke.sk (M.B.); miroslava.badidova@student.tuke.sk (M.B.); 2 Department of Automotive Production, Faculty of Mechanical Engineering, Technical University of Kosice, 040 01 Kosice, Slovakia; janette.brezinova@tuke.sk; 3 Department of Manufacturing Production Engineering, Rzeszow University of Technology, 35-959 Rzeszow, Poland; bcktmiop@prz.edu.pl; 4 U.S. Steel Kosice, s.r.o., 044 54 Kosice, Slovakia

**Keywords:** coated sheet, artificial ageing, salt mist corrosion test, coating adhesion

## Abstract

This contribution presents the results of research focused on changes in selected properties of organic-coated steel sheets in a corrosive environment of salt mist. The aim of this study was to characterise coated sheets and to analyse the influence of elevated temperature on changes in their selected properties. The methodology and experiments were chosen to simulate the accelerated ageing and to evaluate the surface of organic-coated steel sheets. During the research, the effect of artificial ageing due to increased temperature on changes in mechanical properties of coated sheets was monitored, which were determined by tensile testing according to STN EN 10002-1. The fracture surface of the test samples was analysed using a SEM. The following observations were made for the evaluation of coating by cross-cut tests according to EN ISO 2409. Corrosion tests were carried out by conducting salt spray corrosion tests according to EN ISO 9227. The test specimens were artificially sectioned using a laser beam. The results of the research experiments confirmed the assumption that the utilisation of the laser beam was convenient for the creation cross–cut test of the material. After the ageing test, only small significant changes in the basic mechanical properties of the tested materials were observed. It is necessary to take into account that there were changes in the appearance of the coating. The cross-cut test confirmed the results that the plastic-coated sheets could be utilised in salt corrosion environments.

## 1. Introduction

Coated sheets have been intensively researched and developed, especially in Great Britain, Germany, Belgium, and the USA. Some foreign metallurgical companies began producing strips of various widths and lengths, which were unilaterally or bilaterally plasticised, after several years of research, aiming to replace tin. In 1955, 100,000 m^2^ of this material was produced in the USA for consumer industries.

In Europe, one of the first plasticising methods was developed in 1956. At that time, in Great Britain, the prevailing opinion was that direct covering with self-adhesive foil would be best. However, this procedure was later replaced by other methods; abroad, the lamination and plastisol methods gradually gained popularity. In these methods, an adhesive layer is applied to the sheet before plasticising [1].

The method of applying additional coatings to sheets that are galvanised with organic coatings is currently trending. When combining metallic and organic coatings, it forms a combined or duplex coating system. This system provides long-term protection to the steel surface if certain requirements are met.

The main requirement is the necessary interaction between the organic coating and zinc, ensuring adhesion between the base steel, the coating, and the paint. It also prevents the rupture of adhesive bonds between the base steel, the coating, and the paint. It ensures corrosion resistance too. Steel sheets with organic coatings are indispensably used in industrial areas where corrosion resistance is important, especially in the construction, automotive, and consumer industries [2,3,4,5,6].

The basic materials used for manufacturing coated sheets are steel sheets which are either hot-dip-galvanised or cold-rolled without surface treatment [2,3,4,5,6,7]. The lifespan of such treated sheets is a minimum of 40 years under normal conditions. 

**Figure 1:**
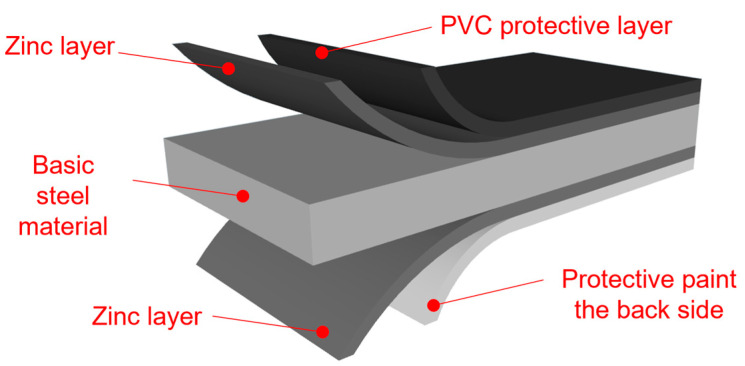
Organic-coated steel sheet.

By combining individual layers, the final properties of coated products are ensured. The manufacturer determines the pre-treatment and primer to ensure the best adhesion between layers. In most cases, a topcoat is applied to the top layer of the sheet, and a backcoat to the bottom layer; however, a topcoat can also be applied to the backside if special product properties are required (e.g., for drips). The coating thickness is a combination of primer and topcoat thicknesses. Usually, the topcoat constitutes the majority of the coating, and the basecoat has a thickness of 5 μm. There are also cases where a thicker basecoat is used, where the primer thickness accounts for half of the total coating thickness [5]. An example of a coated sheet surface-treatment system is illustrated in Figure 1.

According to the study by the European Coil Coating Association (ECCA), the largest growth in coated sheets is expected in the areas of polyurethane and polyester. Regarding the use of PVC coatings, there has been a slight decline due to environmental considerations, but they have remained on the market due to their good properties. Highly durable polyesters have replaced coatings based on polyvinyl chloride [8].

There is increasing demand in the market for pre-treatment processes that are waterless. A coating is applied to the sheet by roller-coating and then it is dried. Therefore, rinsing is not necessary, avoiding the problem of wastewater pollution and its treatment.

Discussions also revolve around the use of sheets with organic coatings under atmospheric conditions. Various tests are being conducted, especially in determining resistance to atmospheric influences, where there are varying degrees of pollution [8,9,10].

In the transportation industry, these sheets still have a place and have been used for many years. Continuously coated sheets ensure corrosion protection, especially in critical areas. The sheets are easily formable, and can be laser-welded, riveted, or glued without issues. Spot welding is problematic, but with an appropriate combination of steel, pre-treatment processes, and paint material, problems can be resolved [11,12,13,14].

The production of coated sheets can be divided as follows:

- Application of finished foils—lamination;
- Application of plastisol;
- Application by swirling finely ground plastic powder in fluidisation chambers;
- Flame spraying (with plastisols, powder materials);
- One-sided or double-sided lamination [1,5,6].

In terms of quality, coated materials differ from traditionally coated materials by two main factors [13,14,15]:

1. The individual layers naturally cooperate (substrate, pre-treatment, paints);
2. The process is highly controlled, ensuring uniformity in cleaning, pre-treatment, and paint application.

**Figure 2:**
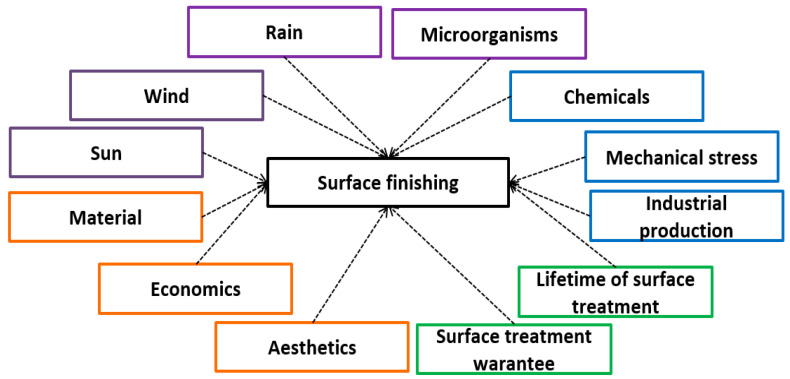
Anti-corrosion protection of steel sheets.

The quality of sheets is significantly affected by the environment, in which they are located or stored. The environment, whether dry, liquid, or humid, and the storage temperature are crucial. The air, or rather its temperature, causes material ageing. Many factors influence the surface treatment of the material, as shown in Figure 2 [14,15,16,17,18,19].

In the case of the surface treatments of materials, including sheets, there are two types of problems:

The first one is ecology. Emissions of various gases and particulate matters significantly pollute the atmosphere. In particular, emissions of SO_2_ cause adverse impacts on the environment overall, such as the devastation of coniferous forests, and on humans, manifesting as respiratory diseases. It also has a negative impact on metal surfaces, significantly accelerating their corrosion. Similarly dangerous pollutants include hydrogen fluoride, hydrogen chloride, and nitrogen oxides [10,18,19]. 

Industrial waters, which contaminate water bodies, have been increasingly polluted with chlorides over the years. This deterioration affects the corrosion resistance of stainless-steel structures where pitting corrosion occurs already at the concentration of 2 mg/L. In continuous production, the quality of rinses after specific chemical or electrochemical operations in steel surface treatments is a significant issue. The presence of dust in the atmosphere has increased by approximately 20% since the beginning of the 20th century. The presence of salts of noble metals, mainly mercury, and the adsorption of aggressive gases on dust grains cause intensive corrosion. Lead from exhaust gases also contributes to this corrosion [18].

The impact of the environment on the surface and properties of coated materials manifests in the corrosion, wear, and degradation of coated sheets. In real life, coated sheets experience wear. 

Wear can be divided according to [18,19,20,21]:

1. Mechanical
  - Metal/metal: oxidative, thermal, abrasive (rubbing), pitting (surface treatment), and combined;
  - Hard particles—abrasive/metal: abrasive bound/metal, abrasive powder/metal, abrasive in liquid-flowing medium—hydraulic erosion, and abrasive in gas-flowing medium/metal;
  - Liquid-flowing medium/metal: turbulent flow of liquid/metal—hydraulic erosion, and flow of liquid and water vapour/metal—cavitation;
  - Gas—flowing medium/metal: pneumatic erosion.
2. Physical–chemical wear,
3. Chemical wear:
  - The nature of the surface—corrosion—can be chemical or electrochemical, namely: under tension, under fatigue, or under vibration;
  - Method of failure—uniform corrosion or uneven corrosion—can be point, spot—local, intercrystalline, and extraction—selection, or by irradiation.

The ability of plastics to resist corrosion is determined by their chemical and molecular structure. The reaction of polymers in crystalline form within corrosive environments is slower than that when they are in an amorphous state. This is because diffusion into plastics containing a crystalline phase proceeds more slowly. 

The results of polymer testing can be significantly influenced by the temperature or the relative humidity. There are three stages in which the environment affects the test results [15,16,17,18,19,20,21,22,23]:

- Storage conditions;
- Conditioning conditions;
- Test conditions.

Corrosion is a physical–chemical interaction between metal and the environment, leading to changes in the properties of the metal, which can often cause deterioration in the function of the metal, the environment, or the technical system. The purpose of corrosion protection is to protect steel from material and economic losses through properly designed surface treatment [16,24,25]. According to the standard EN ISO 12 944 [26], four categories of service life are distinguished:

- Low (L)—up to 7 years;
- Medium (M)—from 7 years to 15 years;
- High (H)—from 15 years to 25 years;
- Very high (VH)—more than 25 years.

The service life of the surface treatment is not a “warranty period”. The service life is a technical assumption (planned parameter) according to which the maintenance plan is drawn up. The warranty period is a presumption agreed upon within the contractual conditions of the relevant commercial agreement between the investor and the contractor. Usually, the warranty period is shorter than the service life. There is no dependency between the warranty period and the service life.

Storage is the time that elapses from the production of the body until the testing process. The conditioning of test bodies is performed to achieve an equilibrium state of the sample under test conditions. The conditioning of polymer materials before testing is necessary to stabilise the properties of the material so that they meet the conditions prescribed for the test. The evaluation of the corrosion resistance of organic coatings can be performed using various methods. Accelerated laboratory tests are commonly used in practice, and in automotive manufacturing, an accelerated laboratory test in a salt mist environment is often used. The resistance of coatings is also evaluated around an artificially mechanically created test cut. Different standards use different shapes of test cuts. The distance of corrosion from this cut provides information about the electrochemical action of the corrosion-inhibiting pigment used in the coating. If there is no corrosion around the cut, then the corrosion-inhibiting pigment used is active in the cathodic or anodic area of corrosion [23,24,25,27].

**Figure 3:**
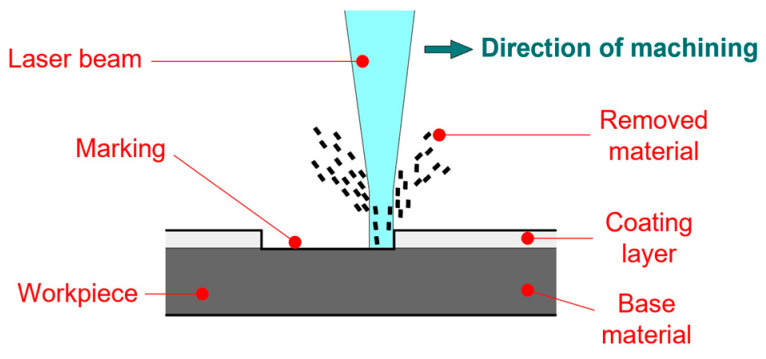
Ablation removal of coating.

**Figure 4:**
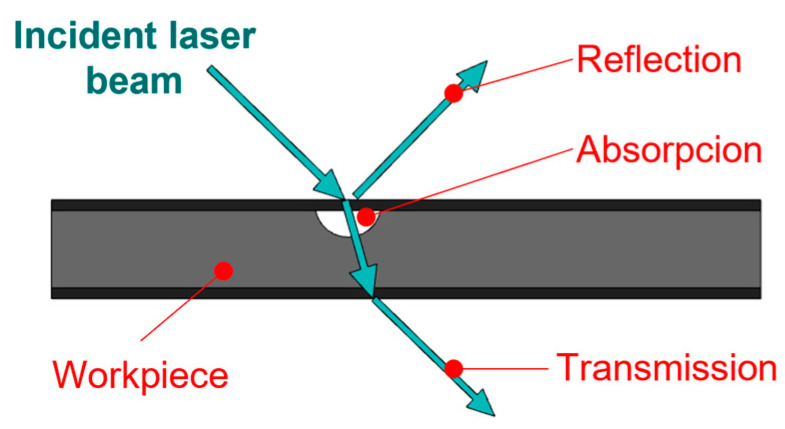
Reflectance and absorption of a laser beam.

**Figure 5:**
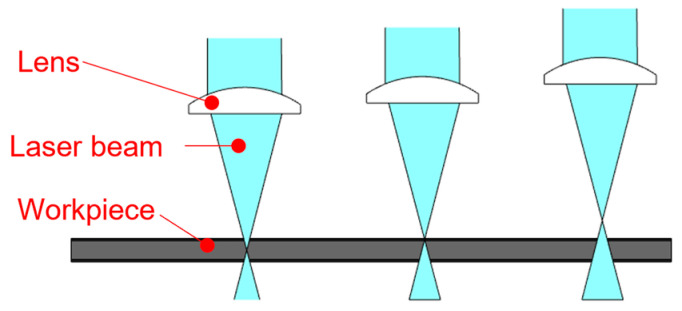
Focusing the laser beam and its impact on an engraved surface.

Various tools can be used to create the test cut. Laser technology is often used in practice for this purpose [28,29,30]. Lasers are part of everyday life in modern manufacturing, including surface-finishing technologies [31,32,33,34,35,36,37,38,39]. With lasers, we can mark or remove layers of foil. Ablation, as shown in Figure 3, involves selectively removing the coating using a laser beam [26,40,41,42,43,44,45,46,47]. The selection of the material, its surface properties, the absorbance, and the reflectivity of the laser beam are crucial, as shown in Figure 4. To obtain a quality cut, it is necessary to focus the beam [31,32,33,34,35,36,37,38], as shown in Figure 5.

## 2. Materials and Methods

For the experimental tests, coated sheets from the production of U. S. Steel Kosice, s.r.o. (Kosice, Slovakia) were used. The experimental work was performed on coated sheets [5] where the base material consisted of high-strength steel sheets, designated DXS1D + Z + ZF, according to EN 10346 [48], with a zinc coating of 225 g/m^2^. A standard polyester was used as the coating.

The coating system of the produced tested material from the producer U.S. Steel Kosice, s.r.o. consisted of 3 layers and was named RAL (according to the producer), which also had various colour scales. The used tested materials were [5,48,49]:

- RAL 9002 Grey–White (white), with a sheet thickness of 0.4 and a paint thickness on the top side of 10 ^+ 5^ μm;
- RAL 8017 Oxide Red (brown), with a sheet thickness of 0.5 and a paint thickness on the top side of 20 ^+ 5^ μm;
- RAL 9006 Standard Polyester (Silver), with a sheet thickness of 0.6 and a paint thickness on the top side of 20 ^+ 5^ μm.

To ensure the testing of coated sheets under consistent conditions, according to the manufacturer [5,48,50], the test samples were conditioned according to the standard ISO STN 558—Conditioning and Testing—Standard Atmospheres—Definitions [51]. The testing of organic coatings is regulated by the standard ISO STN 1110—Plastics—Polyamides—Accelerated Conditioning of Test Specimens [50].

For corrosive conditions in the EU, the standard atmosphere is defined to have a temperature of 23 °C and a relative humidity of 50% (referred to as 23/50) at a pressure of 86–106 kPa. The permitted tolerances are 2 °C for the temperature and ±5% for the relative humidity.

Table 1 shows a comparison of the designation of tensile steel grades according to various standards. Table 2 presents the chemical analysis of the test samples. Additionally, Table 3 lists the mechanical properties of the steel sheet test samples.

The properties of the organic coatings meet the following specifications [3]:

- High resistance to UV radiation—RUV 2;
- Coating thickness (topcoat + primer [μm])—20 ^+ 5^ μm;
- Adhesion (grid test)—0–1.

**Figure 6:**
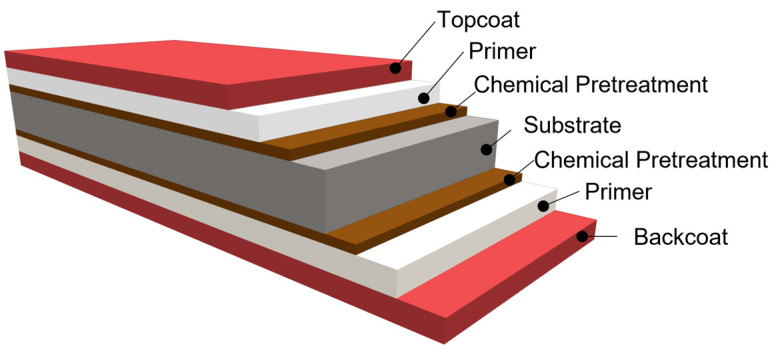
Organic-coated sheet metal-3-layer coating system.

The coating was applied to the sheet by rollers with the desired wet film thickness. Subsequently, it was dried in an oven at a temperature of 220–260 °C [3]. The composition of the coating system is depicted in Figure 6 [3]:

- Conversion intermediate layer (passivation) without chromium content (thus, Cr-free);
- Polyester-based primer with a thickness of 5 μm;
- Topcoat based on polyester with a thickness of 20 μm;
- Backcoat based on polyester with a thickness of 7 μm.

The entire coating system on the sheets is chromium-free.

### 2.1. Experimental Methods

Based on global research and obtained information on the utilisation of the coated steels, the materials were evaluated for application on roofs and fences, where they are exposed to extreme atmospheric conditions and UV radiation. The experimental program was designed to evaluate the changes in mechanical properties and the protective effect of the coating system under simulated operating conditions. 

Our study was oriented and divided into the following steps:

1. Evaluation of chosen mechanical properties and evaluation of coating behaviour of tested plastic coated samples after tensile stress (where we evaluated surface changes, curling of the coatings after the tensile test, and artificial ageing) at normal temperature and after ageing at temperatures of 100 °C and 120 °C;
2. Laser marking of the tested plastic-coated samples, creation of test cut, and evaluation of the plastic coating after laser marking (like burning of the coating and measurement of the distance of the affected zone and destroyed plastic layer). Mechanical test tools were used to create a test cut in practice; a laser beam was used to obtain a smooth test cut through the entire coating system up to the applied material;
3. Evaluation of the tested samples after laser marking (plastic coating sheet samples after laser marking) by metallography and after the salt corrosion test.

#### 2.1.1. Preparation of the Artificial Ageing of Tested Plastic-Coated Samples

**Figure 7:**
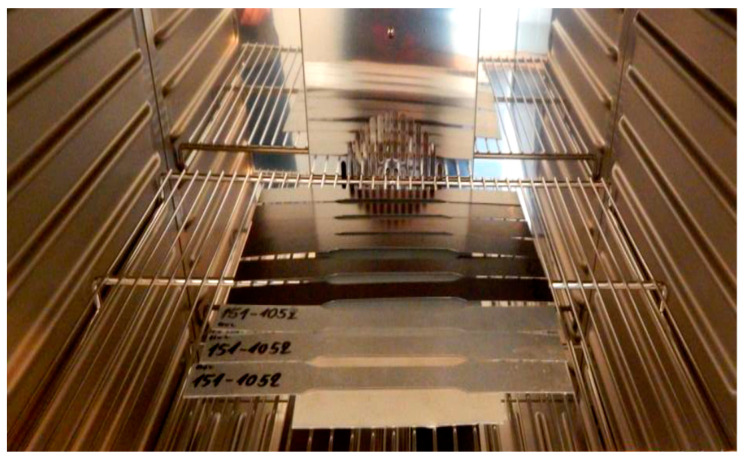
Storage of samples in a laboratory dryer.

The artificial ageing of the tested plastic-coated materials was proposed and carried out in a laboratory-programmable dryer of the brand Memmert UNP 200 with natural airflow, with the fan speed set to 20% power at a temperature of 100 °C for 20 min and 120 °C for 20 min, as shown in Figure 7. The tested samples for ageing were prepared and manufactured for the tensile test according to the standard STN EN10002—Part 1:2001 [54], as shown in Figure 7, and following prescribed standardised dimensions from the mentioned standard, as described in Table 4. 

The Influence of artificial ageing on the changes in the mechanical properties, appearance, and behaviour of the plastic coating was evaluated. 

#### 2.1.2. Static Tensile Testing

**Figure 8:**
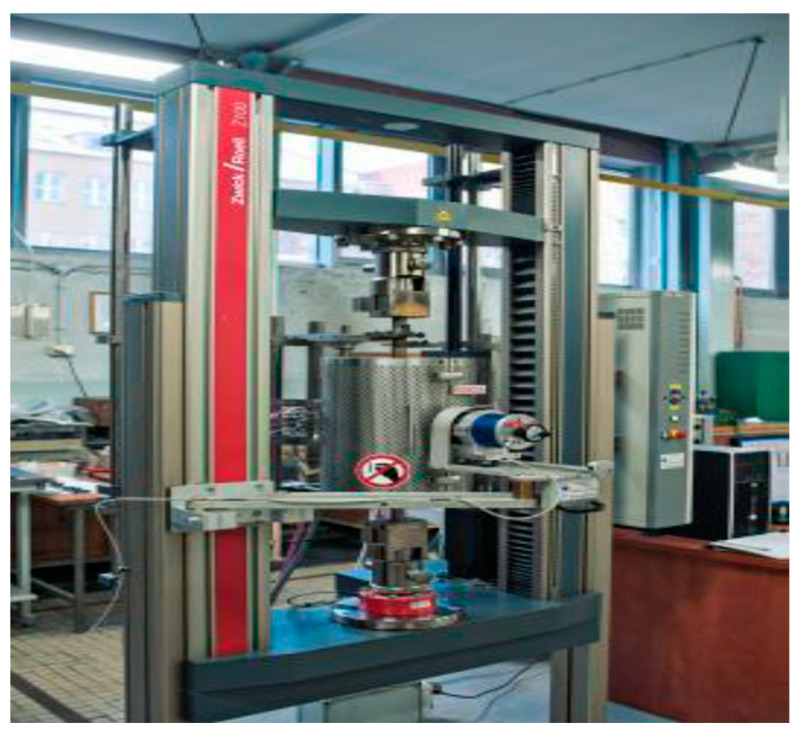
Testing Zwick Z100 shredding machine for experiments.

The tensile test was conducted according to STN EN10002—Part 1 [54] using coated material samples after ageing. The standardised dimension test samples were used for tensile testing on a Zwick-type Z100 tensile testing machine (produced in ZwickRoell, Ulm, Germany), as depicted in Figure 8.

**Figure 9:**
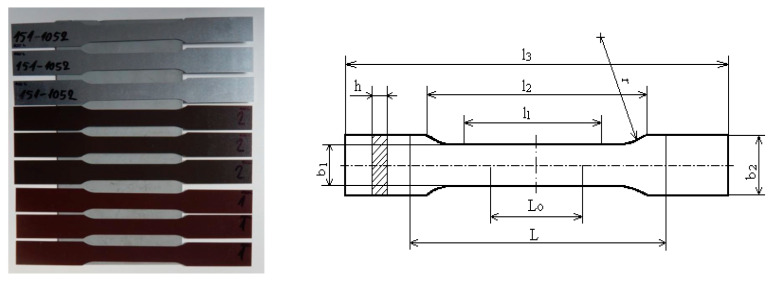
Samples of coated sheets used for ageing and for the subsequent tensile test [54].

In Figure 9, the tested samples of the used coated sheets according to the standard STN EN10002—Part 1 [54], prepared for the tensile test, are shown, while the first 3 samples were only coated with Zn layer without upper coating and the last samples were plastic-coated. The dimensions of the test specimens for the tensile test are shown in Table 4.

#### 2.1.3. Preparation of the Tested Samples for the Laser Cut and for the Salt Corrosion Test

Corrosion is a physical–chemical interaction between metal and the environment, leading to changes in the properties of the metal that can often cause deterioration of the function of the metal, the environment, or the technical system [55,56]. 

**Figure 10:**
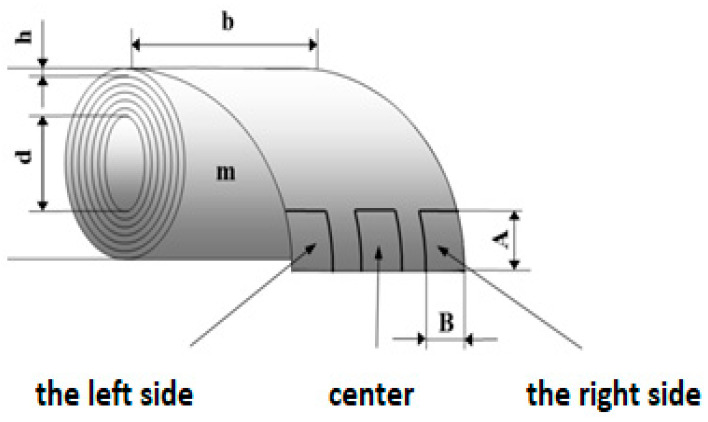
Sampling template from a roll.

**Figure 11:**
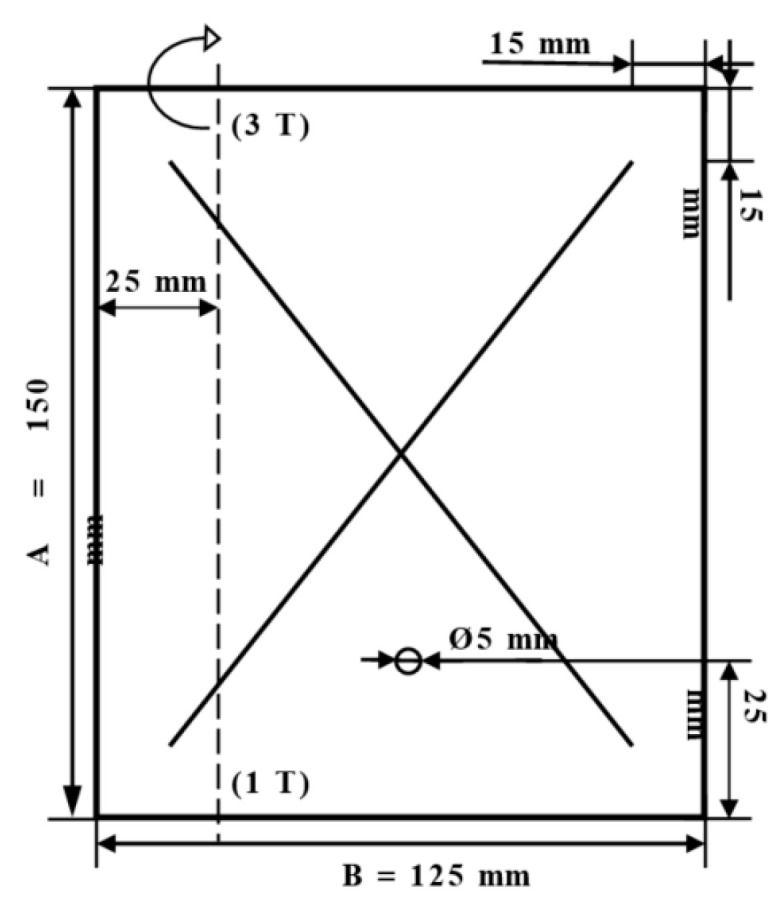
Sample preparation for corrosion tests according to ECCA T8.

**Figure 12:**
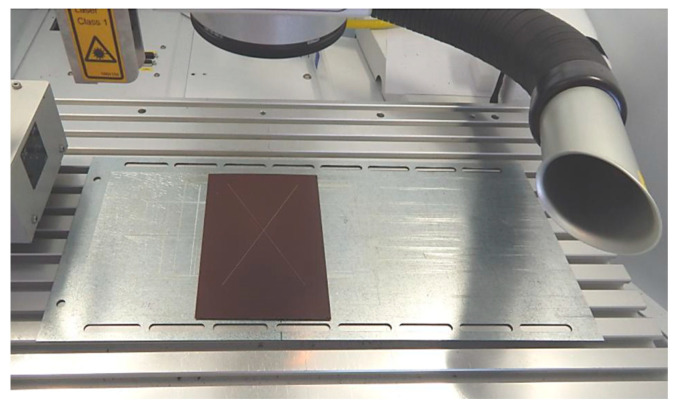
Marking the letter X in the TruMark Station 5000 device.

Before arranging the corrosion test in a salt environment itself, material samples were created, as shown in Figure 10. To determine the presence of under-corrosion, a test cut was made by using a laser beam. The shape of the letter “X” was created in the plastic-coated samples according to the drawing in Figure 11. This process took place at the working hall of TRUMPF Slovakia, s.r.o. (testing laboratory place Košice-Juh, Slovakia), on the TruMark Station 5000 laser device (produced in TRUMPF, Ditchingen, Germany), at a speed of 400 mm/s, a frequency of 28,000 Hz, a beam defocus of 0.00 mm, a power of 100%, and a cycle time of 9.14 s, as depicted in Figure 12.

The purpose of this technology is to introduce laser marking (LM) or cutting technology for plastic-coated materials (PCMs). The method combines the use of laser marking (cutting) during the creation of the shape of the letter X on the tested surface of the plastic-coated sheet and subsequent artificial corrosion testing on it [57,58]. Based on the influence of laser ablation on the absorbing layer of the tested material caused by laser shock, a design experiment was conducted to verify and evaluate the feasibility of obtaining a target-shaped mark using the laser method.

Using the letter X as an example of patterns designed in this paper, the morphology of the mark depended on the “shape-controlling layer”.

Subsequently, the samples were placed in a corrosion chamber manufactured by Liebisch Labortechnik (produced in Bielefeld, Germany), model SKB 400 A-TR. The corrosion chamber is designed to simulate the corrosion process by alternating salt spray and condensation cycles, which are conducted to assess the resistance of surface coatings. Corrosion tests in this chamber were conducted according to the standard ISO EN STN 9227:2006—Corrosion Tests in Artificial Atmospheres; Salt Spray Tests and ISO 9227:2022/Amd 1:2024 [59,60].

**Figure 13:**
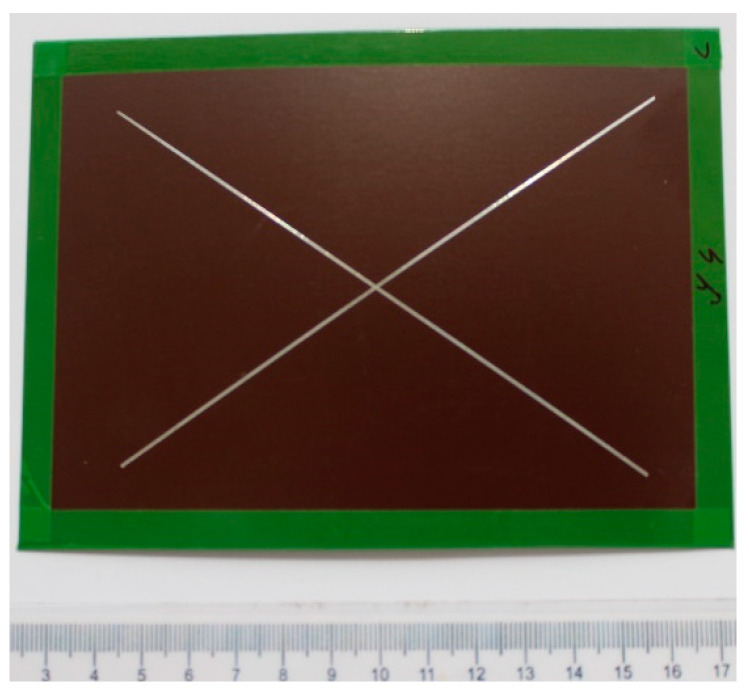
Prepared test sample with X-section.

Figure 13 shows an example of a picture of a test sample with a grid before being inserted into the condensation chamber.

The evaluation of undercutting and the extent of corrosion along the cut, as well as the degree of corrosion under the coating near the cut, was conducted according to the ASTM D 1654-92 method [49]. The undercutting of cut M after exposure to a salt chamber according to EN ISO 12944-6 [57] must not exceed 1 mm.

A laser-type TruMark Station 5000 was used to create the test cut because it was necessary to create a high-quality cut down to the base material through all surface layers (surface system).

The degrees of corrosion attack around the cut are provided in Table 5. 

The result was classified into grades from 0 to 5 according to the relevant standard depending on the extent of corrosion. The grid method was used to assess the adhesion of the organic coatings before and after exposure to the condensation chamber, with the exposure times of samples in the condensation chamber being 240 and 360 h. The corrosion around artificially prepared mechanical damage to the coating by cutting was evaluated. The extent of corrosion attack from this cut provides an indication of the electrochemical action of the anticorrosive pigment used in the coating.

## 3. Results and Discussion

### 3.1. Visual Assessment of Samples after Artificial Ageing

After artificial ageing under laboratory conditions, no visible damage on the surface of the coated tensile test samples was observable to the naked eye.

The colour of the sample surface remained unchanged. There was no damage to the top coating layer, and no peeling, cracking, formation of osmotic blisters, or other damage types occurred.

### 3.2. Tensile Test According to STN EN 10002-1

**Figure 14:**
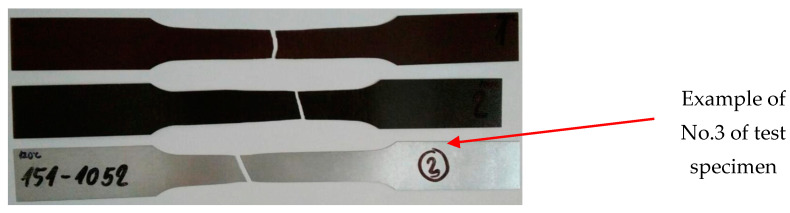
Visual evaluation of the samples after the tensile test.

**Figure 15:**
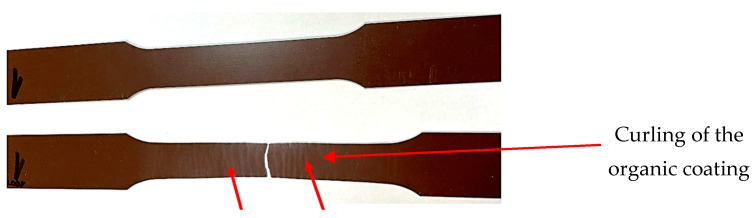
Comparison of sheets before and after the uniaxial tensile test with visible corrugation.

**Figure 16:**
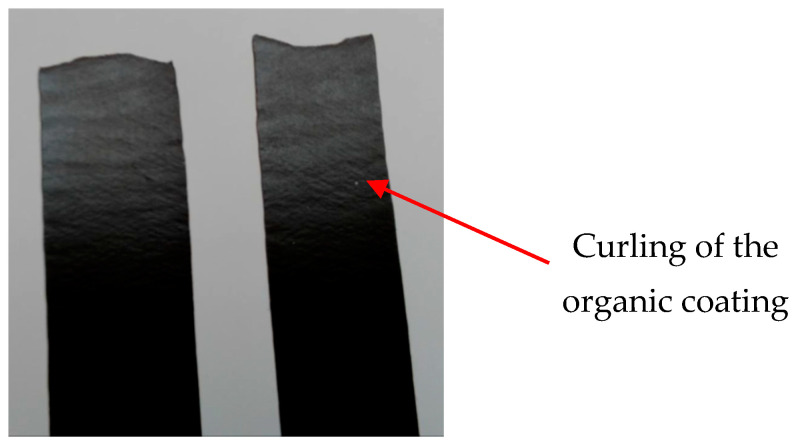
Detail of a torn, aged tensile test specimen at a temperature of 100 °C.

Figure 14 shows the appearance of coil-coated tensile test [54] samples after artificial ageing at 120 °C and after subsequent tensile testing. The breakage occurred in the measured area as recommended by the tensile test standard [54]. Figure 15 compares the test samples before and after tensile testing. Deformation of the rods is visible around the tensile fracture and elongation occurred in the measured part (originally sized at 80 mm), as shown in Figure 16.

In Figure 16, a detail of the torn test sample after ageing (at 100 °C) shows slight material waviness, indicated by an arrow, but there was no damage to or delamination of the coil-coated layer.

The results of the tensile test evaluated the basic mechanical properties, the yield strength (Re), the ultimate tensile strength (Rm), and the elongation at break (A_80_). The values of Rm ranged from 343 to 398 MPa for samples aged at 100 °C and from 344 to 393 MPa for samples aged at 120 °C. All values of the tensile test results are provided in Table 6 and Table 7.

Table 6 shows the average values from five measurements after the tensile test at a laboratory temperature of 20 °C. For the test, organic coating sheets were used after ageing at a temperature of 100 °C for 30 min. The dimensions of the samples marked as width “a”, thickness “b”, and specific length “L” were used in the calculation of Re and Rm. The values of Re_H_ are the upper limit of slip and those of Re_L_ are the lower limit of slip. The value Rp_0.2_ is the measured value at 2% deformation of the tensile sample, A_80_ is the ductility at 80% deformation of the sample, and Ag is the ductility at homogeneous deformation. 

Table 7 shows the results from tensile test of samples with ageing temperature 120 °C.

**Figure 17:**
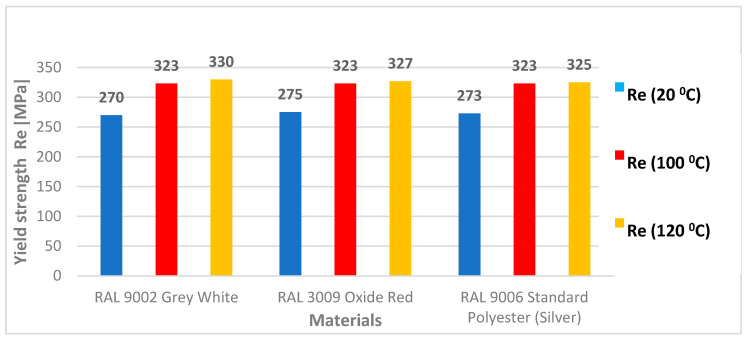
Yield strength values Re of test samples before (20 °C) and after ageing of test samples for 30 min at temperatures of 100 °C and 120 °C.

Figure 17 shows and compares the Re values before ageing (blue colour) and after ageing at 100 °C (red colour) and 120 °C (yellow colour) of three materials (RAL 9002 Grey–White, RAL 3009 Oxide Red, and RAL 9006 Standard). The graph shows that the aged samples had higher strength values by about 50 MPa. However, minimal differences in measured values were observed between individual species.

**Figure 18:**
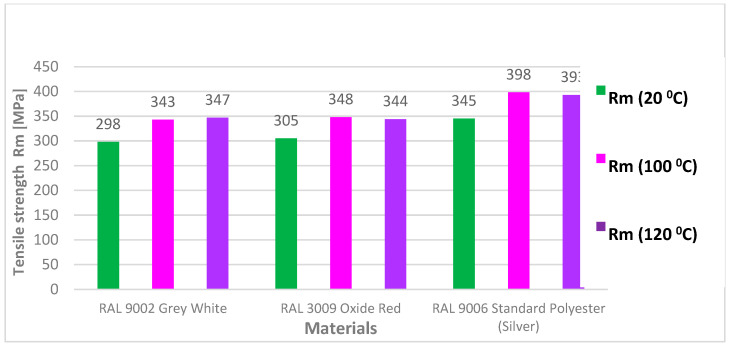
Tensile strength limit values Rm of test samples before (20 °C) and after ageing for 30 min at temperatures of 100 °C and 120 °C.

Figure 18 shows and compares the values of Rm before ageing (green colour) and after ageing at 100 °C (lilac colour) and 120 °C (violet colour) of three materials (RAL 9002 Grey–White, RAL 3009 Oxide Red, and RAL 9006 Standard). The graph shows that aged RAL 9006 Standard samples had the highest Rm values. The differences in values between unaged and aged samples ranged from 45 to 50 MPa. The higher strength values can be attributed to the differences in melting of the materials.

**Figure 19:**
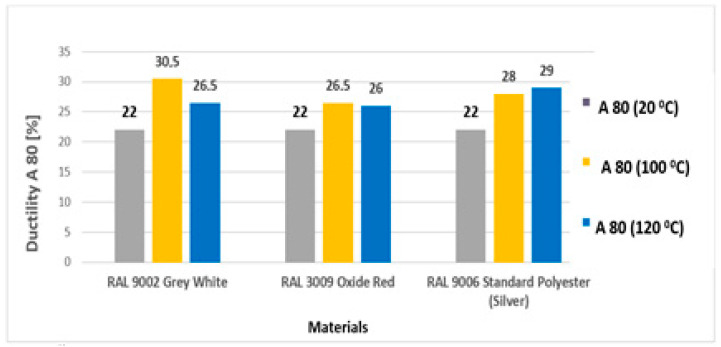
Ductility of samples after the ageing of test samples before ageing and after ageing at 100 °C and 120 °C for 30 min.

In Figure 19, the dependence of material ductility before (RAL 9002 Grey–White) and after the ageing of all types of test samples (yellow and blue) in the laboratory furnace for 30 min is compared.

### 3.3. Metallographic and Microscopic Evaluation of Test Samples

**Figure 20:**
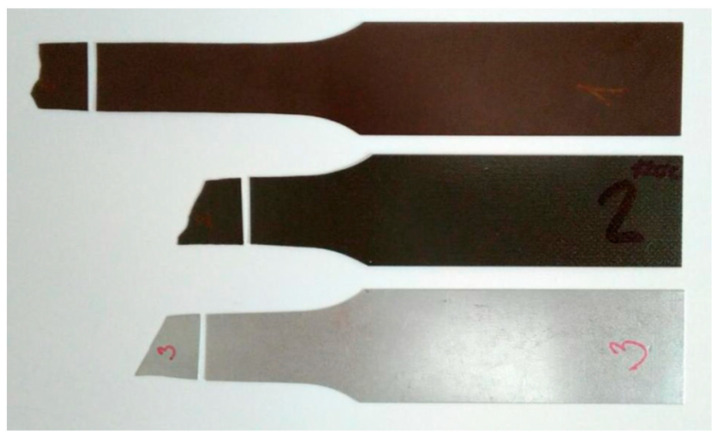
Samples intended for observation under microscopes.

**Figure 21:**
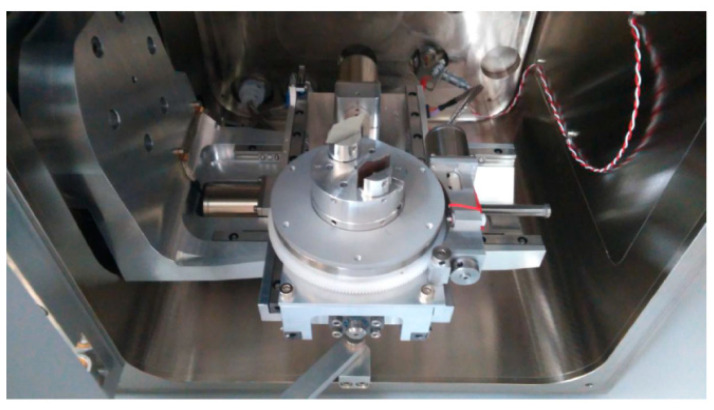
Cut samples stored in a scanning electron microscope (SEM) of type Tescan Vega3.

Metallographic and microscopic evaluation of the test samples was subsequently performed. Microscopic observation of aged and torn samples was conducted using a scanning electron microscope (SEM) of type Tescan Vega3, U.S. Steel, a.s. Košice. Selected test samples of coil-coated sheet metal, aged at 120 °C after tensile testing, were chosen for observation under the microscope. Sub-samples up to 2 cm in size were taken from these samples after tensile testing for observation under the Tescan Vega3 LM microscope, Figure 20 and Figure 21. The Figure 20 shows the samples intended for observation under microscopes. Figure 21 shows cut samples stored in a scanning electron microscope (SEM) of type Tescan Vega3.

**Figure 22:**
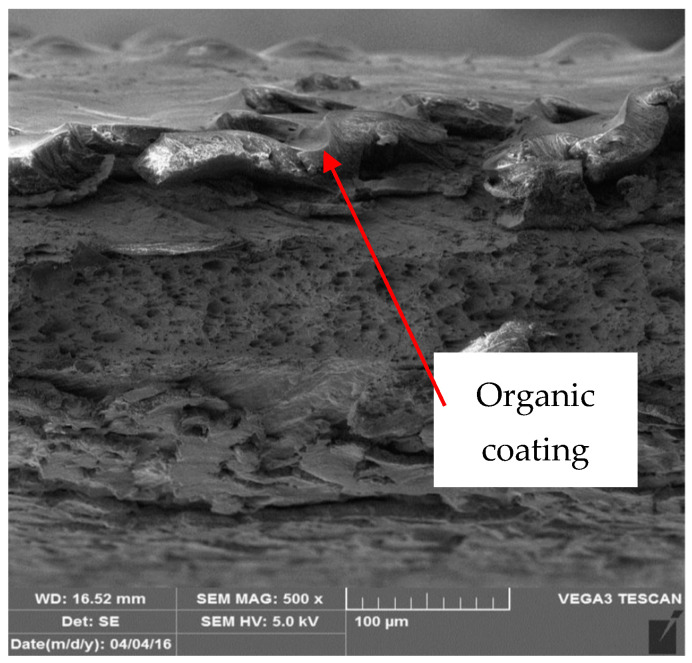
Detailed view of sample organic layer.

**Figure 23:**
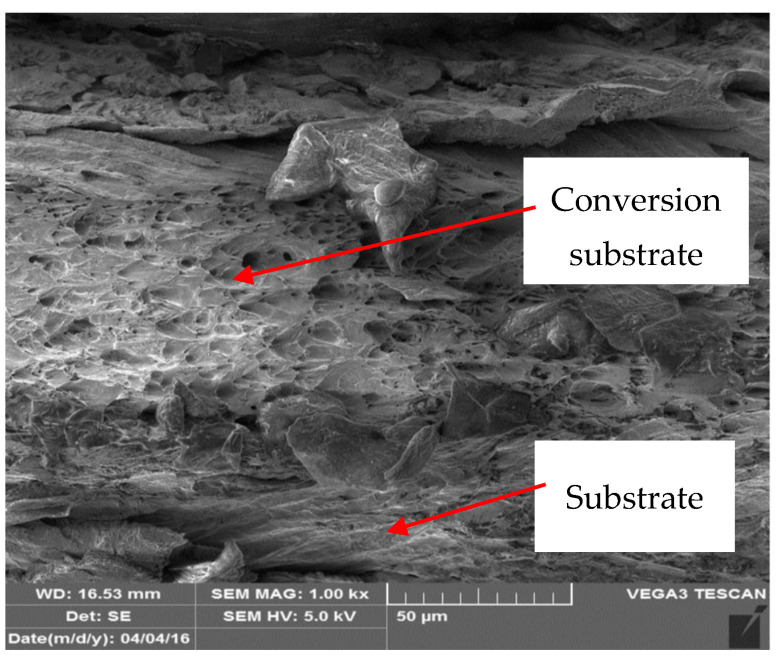
Detailed view of the conversion substrate of the sample.

**Figure 24:**
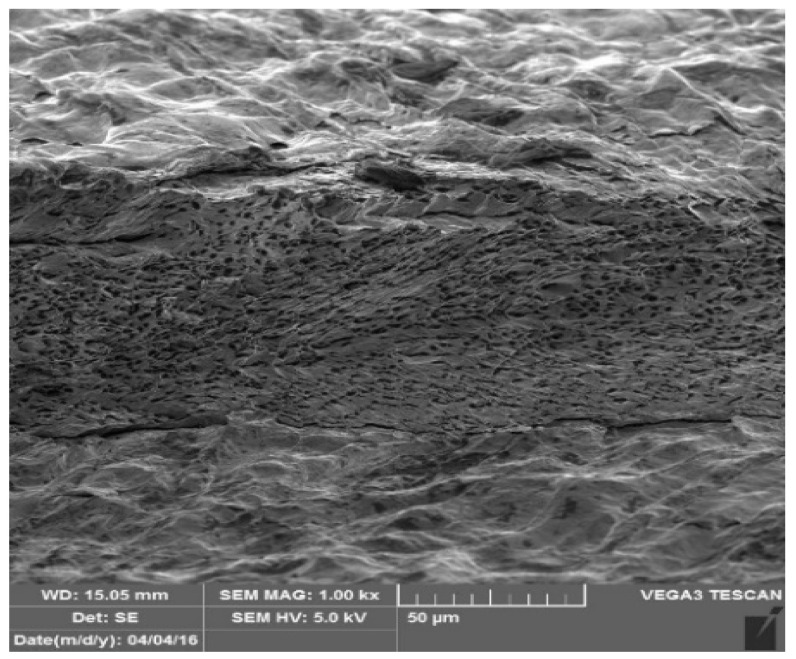
Test sample magnified 500×.

**Figure 25:**
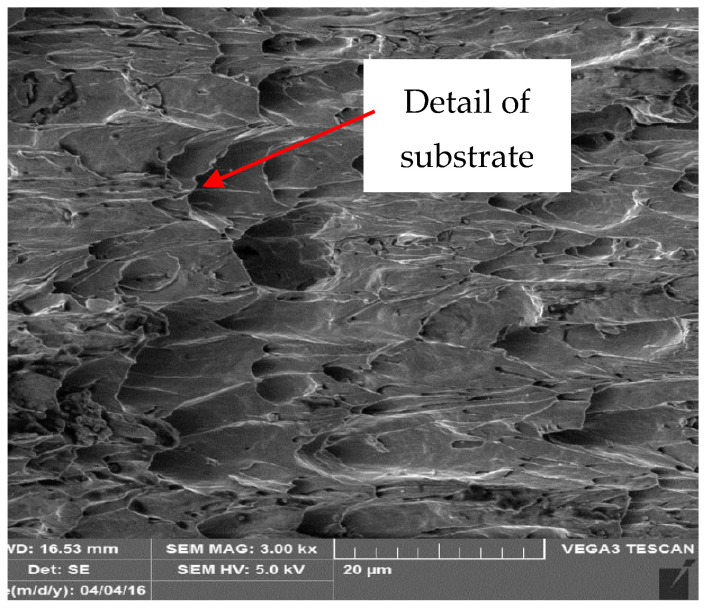
Test sample magnified 1000×.

Figure 22, Figure 23, Figure 24 and Figure 25 illustrate the cut samples of brown coil-coated sheet metal, after ageing and tensile testing, observed under the scanning electron microscope. From a micro-morphological perspective, it exhibited a ductile, dimpled fracture surface. The dimples were elongated in the direction of the applied tensile force.

A light microscope (OLYMPUS GX71, U.S. Steel, s.r.o. Kosice), was used for this purpose.

**Figure 26:**
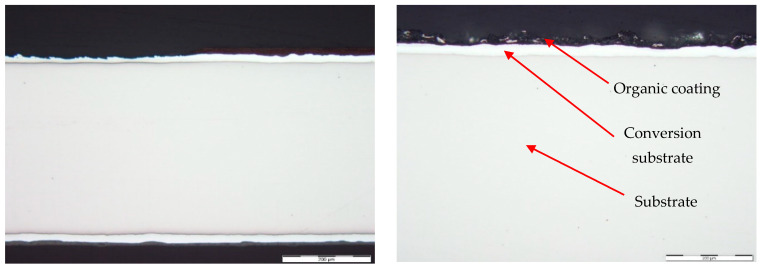
Sample of coated sheet metal in cross-section.

**Figure 27:**
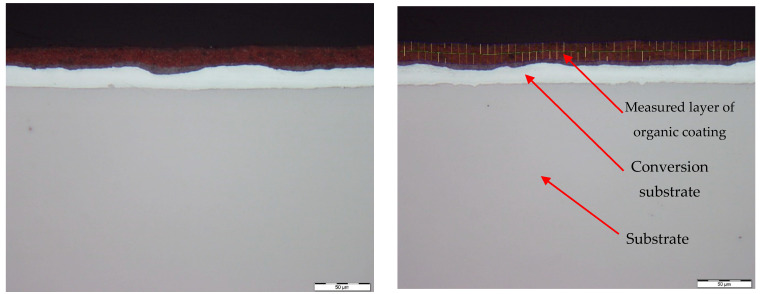
Sample of organic coating and its thickness—brown colour.

Figure 26 and Figure 27 show images of the coil-coated sheet metal in cross-sections before testing, revealing the individual layers of the organic coating, intermediate layers—zinc coating, and the base material—steel sheet. The thickness of the individual layers of the coil-coated sheet metal was also measured.

**Figure 28:**
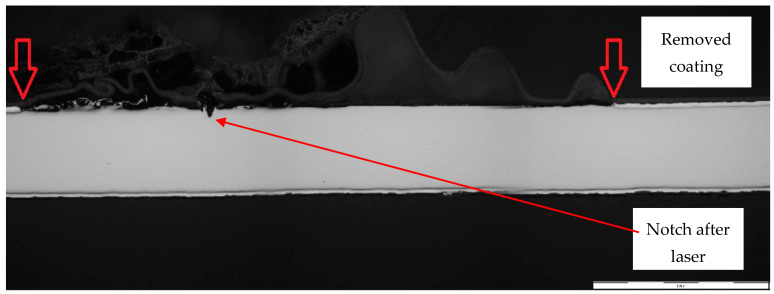
Metallographic section before corrosion testing.

**Figure 29:**
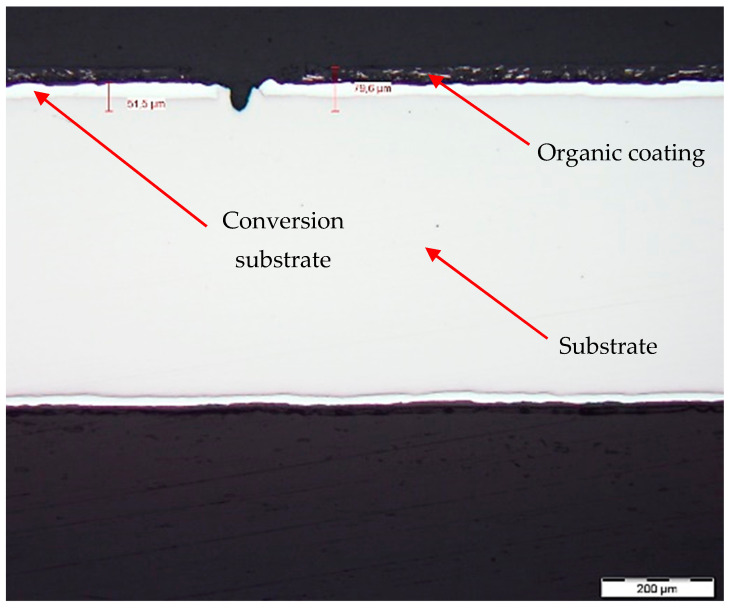
Micrograph of the scratch in the base material and removed coating around the cut.

Figure 28 and Figure 29 show micrographs of the white coil-coated sheet metal at 100× magnification after creating a scratch using a laser beam. The image displays the removal of the coating (space between the arrows) and a scratch in the shape of a “V”. It is evident that the laser scratch penetrated all the way to the base material—directly into the sheet metal. The measured values of the respective depths were as follows:

- Up to a depth of 81.3 μm from the top surface of the sheet metal—from the primary paint to the steel;
- Up to a depth of 56.2 μm from the conversion intermediate layer to the steel;
- Reaching a depth of 40.0 μm into the steel.

Chemical composition analysis of the material layers is essential for experimental tests, as it relates to the properties retained by the layers.

Fractographic analysis of the fracture area was performed and qualitative chemical analysis of different layers was performed using a TESCAN VEGA 3 scanning electron microscope (produced in TESCAN, Brno, Czech Republic) coupled with an OXFORD Instruments EDS analyser (produced in Abingdon, UK).

This is one of the most commonly used methods for determining the chemical composition of thin layers, including the investigated coil-coated sheets.

The chemical composition was measured using the AES method with ARL 4460-513 equipment (produced in Thermo Fisher Scientific, Waltham, MA, USA).

**Figure 30:**
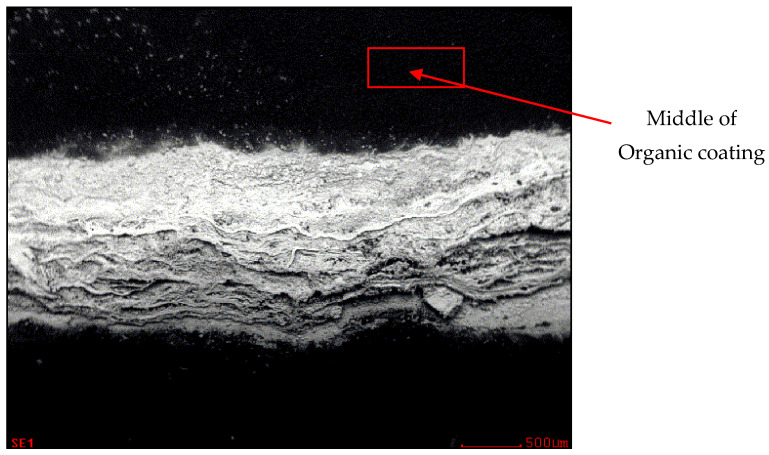
Area of evaluation of the sample—organic coating.

**Figure 31:**
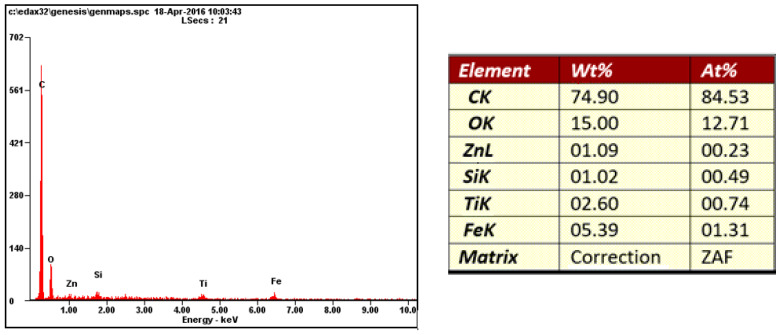
Graph of the EDX spectrum on the sample—organic part—and % content of elements.

**Figure 32:**
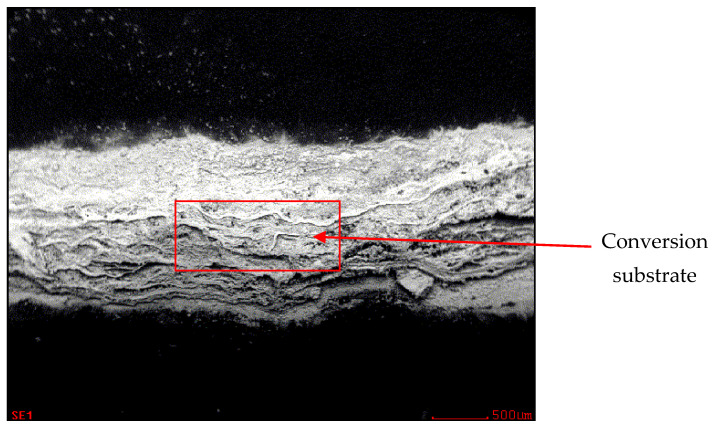
Area of evaluation of the zinc layer of the sample.

**Figure 33:**
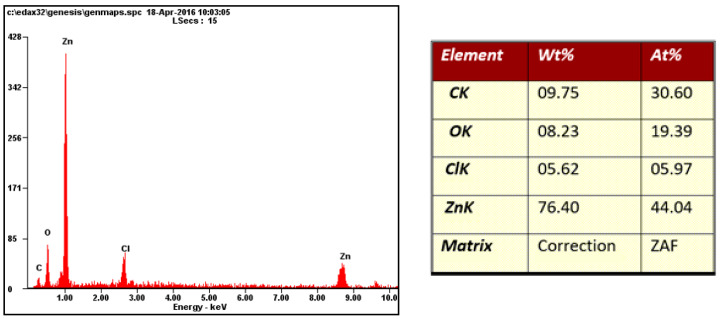
EDX analysis of the zinc layer.

Samples of white coil-coated sheet metal were used for this measurement (Figure 30, Figure 31, Figure 32 and Figure 33). The initial values were measured from the middle area of the plastic coating, where no corrosion occurred, marked with a red rectangle in Figure 30, and an analysis of the organic coating was performed, as shown in Figure 31.

In Figure 32, the zinc layer area is shown, and the chemical analysis of the zinc layer is shown in Figure 33.

The highest proportion of the surface which was undamaged and subsequently exposed to the corrosion chamber, consisted of carbon, followed by titanium, silicon, aluminium, and zinc.

During microscopic analysis, test samples with a cross-cut in the shape of the letter X, created by a laser beam, were evaluated.

The evaluation process involved initially assessing images at 50× magnification, where the thermally affected area of the organic coating and the cut into the base material before the corrosion test were visible.

**Figure 34:**
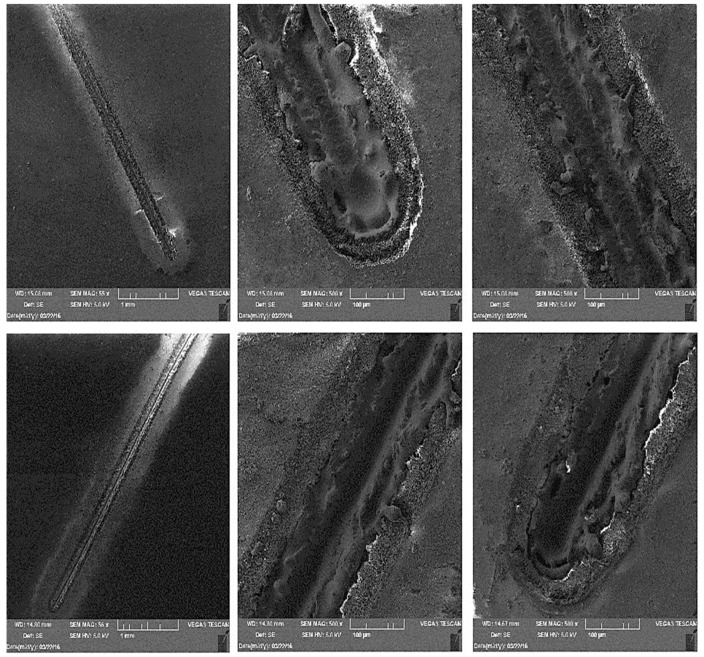
RAL 8017 Oxide Red (brown) test sample and its details after laser processing—cross-cut X.

Figure 34 illustrates the procedure of the creation and evaluation of images of the RAL 8017 Oxide Red (brown) organic coating. Initially, the entire length of the cut was photographed at 50× magnification, followed by detailed images at 500× magnification from various points along the test sample, progressing from the edge to the centre and then to the edge again.

**Figure 35:**
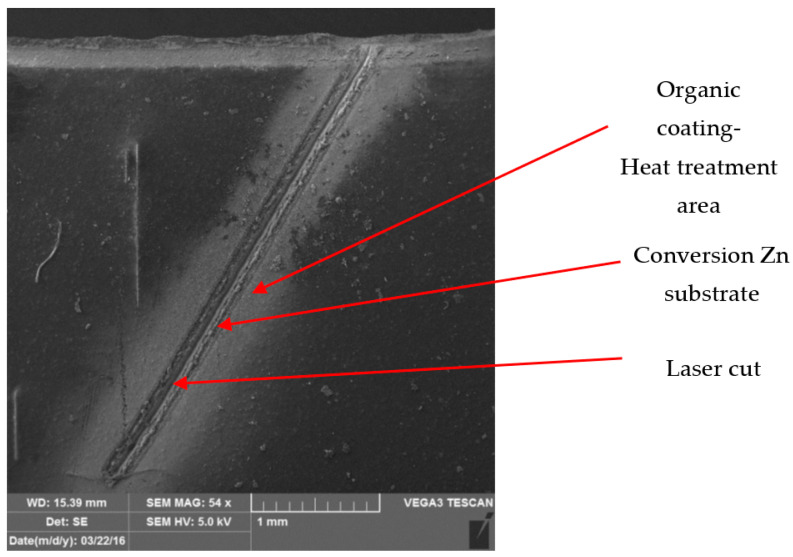
Part of cross-section and heat-affected area after laser processing.

**Figure 36:**
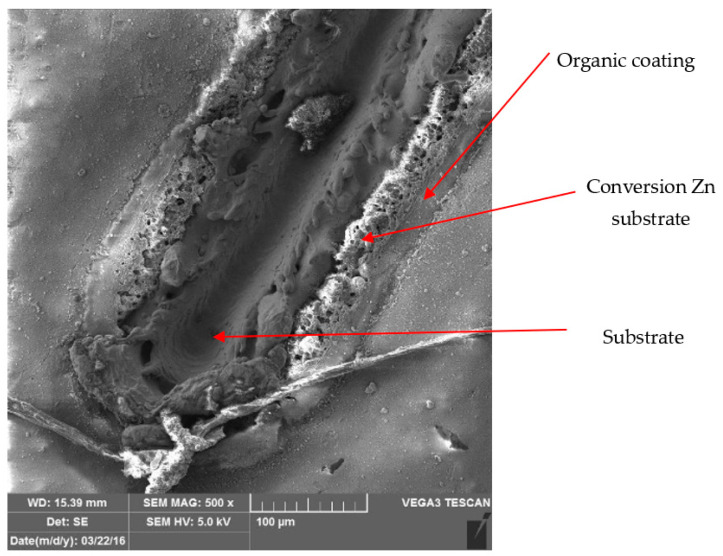
Detail of the end after cross-cutting with a laser beam, vol. 500 times.

**Figure 37:**
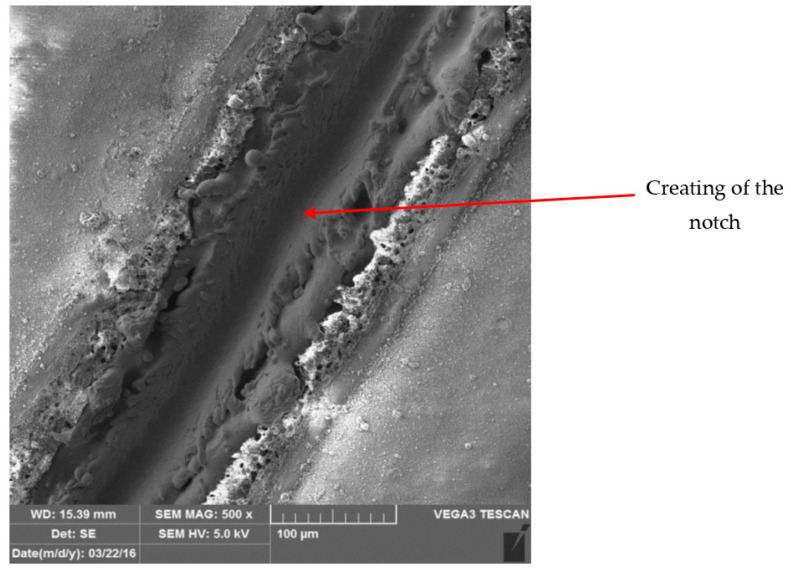
Detail of the middle part of the sample after engraving with a laser beam, vol. 500 times.

**Figure 38:**
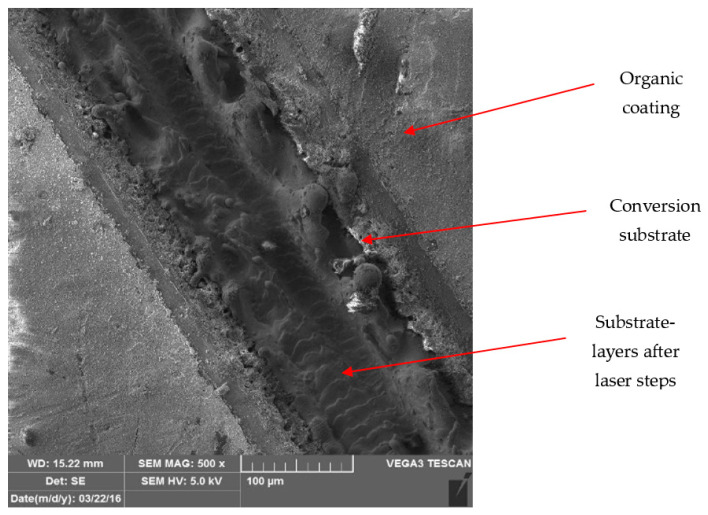
Detail of the movement of the laser beam in the centre of the fused cross section, vol. 500 times.

Subsequent images, Figure 35, Figure 36, Figure 37 and Figure 38, depict images of test samples with RAL 9002 Grey–White organic coating. The images show the length of the cross-cut at 50× magnification where the damaged organic coating and its thermally affected area, melted zinc layer, and base material are visible, followed by images at 500× magnification from different areas of the test sample.

### 3.4. Evaluation of the Corrosion Test

**Figure 39:**
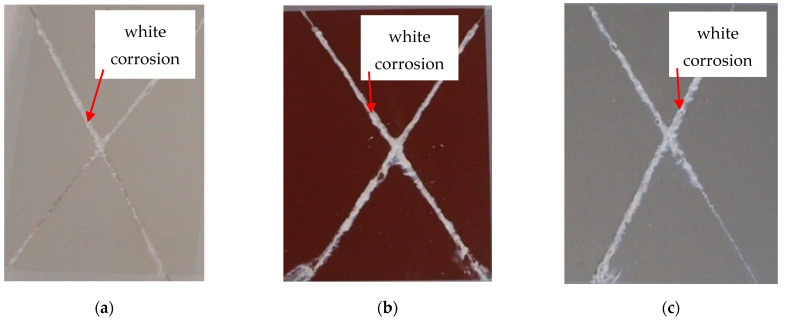
Appearance of test samples after corrosion tests after 240 h: (**a**) RAL 9002 Grey–White, (**b**) RAL 8017 Oxide Red (brown), (**c**) RAL 9006 Standard Polyester (Silver).

**Figure 40:**
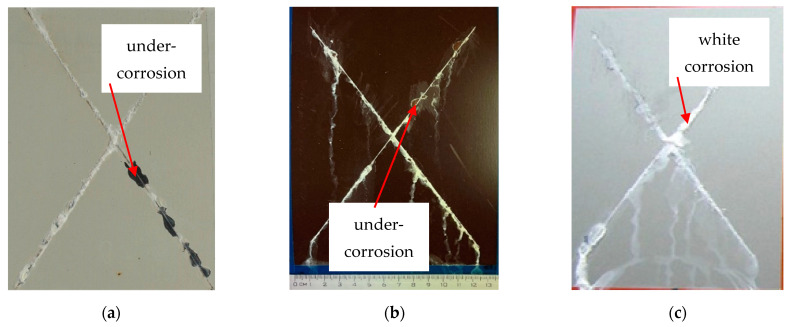
Appearance of test samples after corrosion tests after 360 h: (**a**) RAL 9002 Grey–White, (**b**) RAL 8017 Oxide Red (brown), (**c**) RAL 9006 Standard Polyester (Silver).

Images of the surface of RAL 9002 Grey–White, RAL 8017 Oxide Red (brown), and RAL 9006 Standard Polyester (Silver) coil-coated sheet metal after creating a cross-cut in the shape of the letter X following corrosion tests are shown in Figure 39 and Figure 40. The samples were subjected to simulated corrosion processes for 240 and 360 h in the corrosion chamber. Damage to the material after 240 h, such as layer burning and melting of the base material, and bubble formation, is visible. Views of the test samples after:

(a) Exposure after 240 h;

A smaller range of corrosion products can be observed on the test samples after 240 h; a different corrosion layer could be observed on the white and the silver samples.

On the evaluated samples after 240 h, the so-called “White corrosion“—corrosion products of the Zn layer—was observed. Cathodic protection with a sacrificial anode was confirmed, where, despite mechanical damage to the coating, corrosion of the base material was not observed.

(b) Exposure after 360 h;

Based on the visual assessment of the samples after corrosion tests, differences in corrosion along the cross-cut are visible. The test samples after 240 h in the corrosion chamber exhibited a white corrosion layer. However, after 360 h in the corrosion chamber, the test samples corroded all the way to the base material, showing brown corrosion. 

The extent of the corrosion around the test cut and the degree of corrosion beneath the coating near the cut was determined according to the standard ASTM D 1654-92 method. 

After exposure to a salt chamber according to the standard EN ISO 12944-6, the test samples were also categorised and the group to which each test sample belonged after corrosion was determined. 

After 240 h in a salt chamber, the white and silver samples were classified into group 1 and brown samples into group 2.

After 360 h in a salt chamber, the testing samples were included:

- In the case of silver test samples, the volume of the corrosion products of zinc increased, but there was no corrosion of the base material. They were classified into group 2;
- In the case of brown test samples, there was peeling of the coating in the vicinity of the test section and the corrosion of the base material was recorded. The brown test samples were classified into group 3;
- In the case of white test samples, there was peeling of the coating around the test cut, but no corrosion of the base material was recorded. The white test samples were classified into group 3.

## 4. Conclusions

The aim of this work was to characterise coated sheets and to analyse the effect of increased temperature on the change in their selected properties. The objective of the experimental measurements was to determine the changes in selected properties of the organic-coated sheets in the corrosive environment of salt mist. A procedure for simulating the accelerated ageing process of organic material without corrosion of the basic volume of zinc product-coated steel sheets was proposed. The effect of artificial ageing by the effect of the elevated temperature on the change in the mechanical properties of varnished sheets was determined by a uniaxial tensile test, and the fracture surface of the samples after the test was analysed. 

The adhesion of the coatings was evaluated with an artificially created X-shaped test cut before and after corrosion tests under artificial atmospheres—a test in salt mist. The artificial test cut was made on the test samples using a laser beam.

Based on the experiments, it can be concluded that:

1. After artificial ageing in laboratory conditions, no damage was visible on the surface of the organic coated samples and the colour of the surface of the samples remained unchanged. After the uniaxial tensile test, the Re and Rm values of the ageing samples increased as it was suggested. Due to the influence of testing, the mechanical properties of the test samples did not change significantly; the difference was in the range of 40–50 MPa. By visual evaluation of the tensile test samples, the degradation of the coatings was observed, which was manifested by the curling of the coating after tearing the sample;
2. The corrosion resistance of the evaluated coating systems made of organic coating materials and a Zn layer was sufficient after the corrosion tests; cathodic protection was also confirmed in the place of the artificially created test section. The corrosion resistance of the coatings in the place of the artificially created test cut after 240 h was evaluated in a salt mist environment. After 240 h, the formation of corrosion products—white corrosion of the Zn layer—on the base material was observed. After 360 h, corrosion of the underlying base material also appeared in the RAL 8017 Oxide Red (brown) samples. The cathode protection on the test samples was sufficient for the colours RAL 9002 Grey–White and RAL 9006 Standard Polyester (Silver);
3. For the creation of artificial cuts on the coating, it is possible to recommend the use of a laser; the penetration was up to the base material through the entire coating system.

## Figures and Tables

**Table 1:** Comparison of tensile steel qualities and technical specification—drawing grades [5].

European Union EN 10346/09	Japan JIS G3302 [52]	USA EN 10346/09 ASTM	U.S. Steel Kosice TN USSK 10346-4/13 [53] ZINKOMAGTM
DX51D +Z + ZF	SGC C SGC D1	A 526, A 527, A 528 A 653 CS Type A, B, C	DX51D + Z + ZF

**Table 2:** Chemical composition of test samples [5].

Grade According to EN 10346/09 U.S. Steel Kosice	Chemical Composition (wt. %)					
	C_MAX_	Mn_MAX_	P_MAX_	S_MAX_	Si_MAX_	Ti_MAX_
DX51D + Z + ZF	0.18	1.20	0.12	0.045	0.50	0.30

**Table 3:** Mechanical properties of test samples [5].

Grades According to EN 10346/09 U.S. Steel Košice	Rp0.2 (MPa)	Rm (MPa)	A MIN (%) Lo = 80 mm	r90 min.	n90 min.
DX51D + Z + ZF	-	270–500	22	-	-

**Table 4:** Dimensions of the test specimens for the tensile test [54].

Body Type	Marking	Prepared by Mechanical Processing
Total length (mm)	l_3_	≥150 (200)
Length of narrowed parallel part (mm)	l_1_	80 ^± 2^
Radius (mm)	r	20–25
Spacing between wide parallel parts (mm)	l_2_	104–113
Width of the clamping part (mm)	b_2_	30 ^+ 0.2^
Width of narrowed parallel part (mm)	b_1_	18 ^+ 0.2^
Recommended thickness (mm)	h	1 ^+ 0.2^
Initial measured length (mm)	L_0_	60 ^+ 0.5^
Initial distance of jaws (mm)	L	115 ^+ 1^

**Table 5:** Corrosion attack around the cut according to ASTM D 1654-92 [49].

Degree	Corrosion Area
1 very small	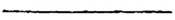
2 small	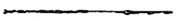
3 a middle	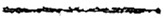
3 b middle	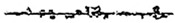
4 a significant	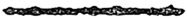
4 b significant	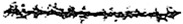
5 very significant	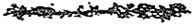

**Table 6:** Tensile test results according to STN EN10002—Part 1 with materials after ageing at 100 °C and at a laboratory temperature of 20 °C.

Samples Number	Ageing Temperature (°C)	Sample Dimensions (mm)			Re_H_ (MPa)	Rp_0.2_ (MPa)	Re_L_ (MPa)	Rm (MPa)	A_80_ (%)	Ag (%)
		a_0_	b_0_	c_0_						
1	at 100 °C	0.496	20	80	356	332	323	343	30.5	18.5
2	at 100 °C	0.493	20	80	364	328	323	348	26.5	7.9
3	at 100 °C	0.413	20	80	326	324	323	398	28.0	17.2

**Table 7:** Tensile test results according to STN EN10002—Part 1 with materials after ageing at 120 °C and at a laboratory temperature of 20 °C.

Samples Number	Ageing Temperature (°C)	Sample Dimension (mm)			Re_H_ (MPa)	Rp_0.2_ (MPa)	Re_L_ (MPa)	Rm (MPa)	A_80_ (%)	Ag (%)
		a_0_	b_0_	c_0_						
1	at 120 °C	0.496	20	80	358	334	330	347	26.5	9.0
2	at 120 °C	0.498	20	80	371	338	327	344	26.0	8.9
3	at 120 °C	0.417	20	80	339	336	325	393	29.0	17.9

## Data Availability

The original parts of contributions presented in the study are included in the article, further inquiries can be directed to the corresponding authors.
